# CRISPR/Cas9 Genome Editing Reveals That the Intron Is Not Essential for *var2csa* Gene Activation or Silencing in *Plasmodium falciparum*

**DOI:** 10.1128/mBio.00729-17

**Published:** 2017-07-11

**Authors:** Jessica M. Bryant, Clément Regnault, Christine Scheidig-Benatar, Sebastian Baumgarten, Julien Guizetti, Artur Scherf

**Affiliations:** a Unité de Biologie des Interactions Hôte-Parasite, Institut Pasteur, Paris, France; b INSERM U1201, Paris, France; c CNRS ERL9195, Paris, France; Washington University School of Medicine

**Keywords:** CRISPR/Cas9, *Plasmodium falciparum*, antigenic variation, transcriptional regulation, *var* genes

## Abstract

*Plasmodium falciparum* relies on monoallelic expression of 1 of 60 *var* virulence genes for antigenic variation and host immune evasion. Each *var* gene contains a conserved intron which has been implicated in previous studies in both activation and repression of transcription via several epigenetic mechanisms, including interaction with the *var* promoter, production of long noncoding RNAs (lncRNAs), and localization to repressive perinuclear sites. However, functional studies have relied primarily on artificial expression constructs. Using the recently developed *P. falciparum* clustered regularly interspaced short palindromic repeats (CRISPR)/Cas9 system, we directly deleted the *var2csa P. falciparum* 3D7_1200600 (Pf3D7_1200600) endogenous intron, resulting in an intronless *var* gene in a natural, marker-free chromosomal context. Deletion of the *var2csa* intron resulted in an upregulation of transcription of the *var2csa* gene in ring-stage parasites and subsequent expression of the PfEMP1 protein in late-stage parasites. Intron deletion did not affect the normal temporal regulation and subsequent transcriptional silencing of the *var* gene in trophozoites but did result in increased rates of *var* gene switching in some mutant clones. Transcriptional repression of the intronless *var2csa* gene could be achieved via long-term culture or panning with the CD36 receptor, after which reactivation was possible with chondroitin sulfate A (CSA) panning. These data suggest that the *var2csa* intron is not required for silencing or activation in ring-stage parasites but point to a subtle role in regulation of switching within the *var* gene family.

## IMPORTANCE

*Plasmodium falciparum* is the most virulent species of malaria parasite, causing high rates of morbidity and mortality in those infected. Chronic infection depends on an immune evasion mechanism termed antigenic variation, which in turn relies on monoallelic expression of 1 of ~60 *var* genes. Understanding antigenic variation and the transcriptional regulation of monoallelic expression is important for developing drugs and/or vaccines. The *var* gene family encodes the antigenic surface proteins that decorate infected erythrocytes. Until recently, studying the underlying genetic elements that regulate monoallelic expression in *P. falciparum* was difficult, and most studies relied on artificial systems such as episomal reporter genes. Our study was the first to use CRISPR/Cas9 genome editing for the functional study of an important, conserved genetic element of *var* genes—the intron—in an endogenous, episome-free manner. Our findings shed light on the role of the *var* gene intron in transcriptional regulation of monoallelic expression.

## INTRODUCTION

The pathogenesis of *Plasmodium falciparum*, the most virulent malaria parasite, relies on the expression of a family of clonally variant adhesion proteins on the surface of infected red blood cells (iRBCs) (1). These proteins, which are members of the *P. falciparum* erythrocyte membrane protein 1 (PfEMP1) family, are encoded by a family of ~60 *var* virulence genes. Similarly to other parasites, *P. falciparum* uses clonal antigenic variation to evade the immune system and to maintain an infection. Thus, only a single *var* gene is expressed at a time, but occasional switching occurs to allow expression of a different PfEMP1 (2). Monoallelic expression of *var* genes is believed to be under epigenetic control, as histone H3 lysine 9 trimethylation (H3K9me3) and H3K36me3 are enriched in the heterochromatin of silent *var* genes whereas transcriptionally permissive histone modifications such as H3K4me3 and H3K9ac are enriched at the single active *var* gene (3–5). It is unclear how one specific *var* gene is targeted for transcriptional activation while all others are maintained in a silent state; however, evidence suggests that underlying genetic elements play a key role in this regulation (reviewed in reference 6).

**FIG 1:**
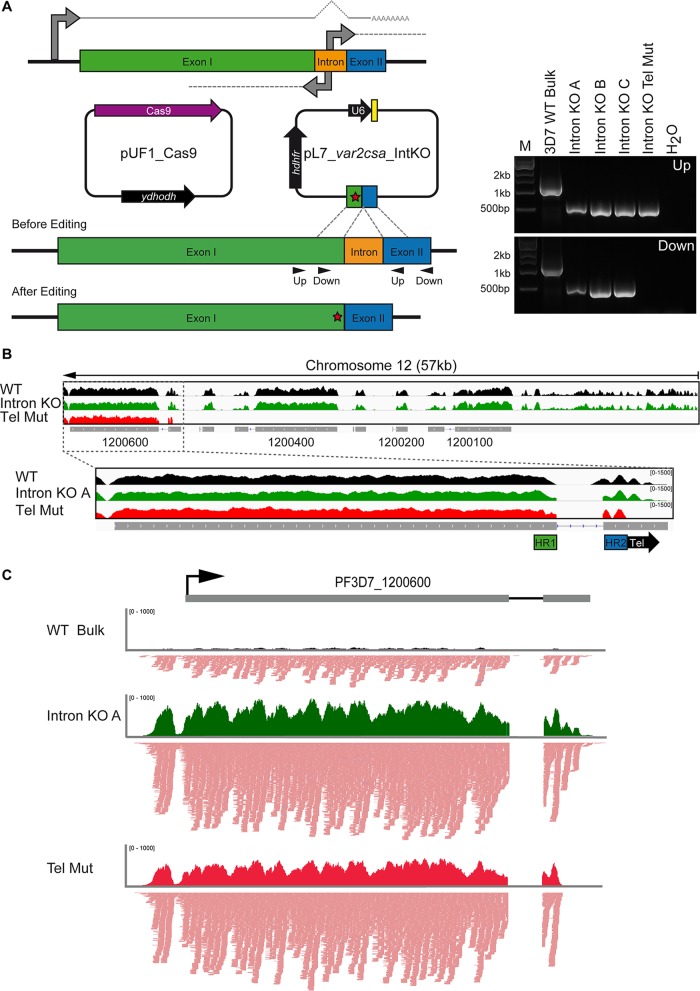
Generation of the intronless *var* gene mutant. (A) Schematic of a representative *var* gene and all associated RNA: full-length mRNA originating from the 5′ promoter and sense and antisense sterile transcripts originating from the intron (solid gray and dotted gray lines, respectively). *var2csa* intron knockout mutants were created by transfection with the pUF1_Cas9 and pL7_*var2csa*_IntKO plasmids. The pL7 plasmid contains the guide RNA (yellow) and DNA homology region KO construct corresponding to the 500 bp of exon I (green) immediately upstream of the intron (orange) fused in frame to the homology region corresponding to the 500 bp of exon II (blue) immediately downstream of the intron. A shield mutation (red) is included in the KO construct. Homologous recombination of the pL7 KO construct with the *var2csa* gene results in the deletion of the intron. Gel electrophoresis of PCRs performed on genomic DNA from 3D7 WT bulk culture (3D7 WT Bulk), three *var2csa* intron KO mutant clones (Intron KO A, Intron KO B, and Intron KO C), and one *var2csa* intron KO telomere mutant clone (Intron KO Tel Mut) demonstrated deletion of the intron. Primers (shown at left as arrowheads labeled “Up,” and “Down”) were designed to detect the presence of the *var2csa* intron. The marker is indicated (M), and a PCR using only water (H_2_O) served as a control. (B) Next-generation sequencing of genomic DNA from WT 3D7 parasites (black), *var2csa* intron KO clone A (green), and a telomere mutant clone (red) showed that the intron was deleted successfully with CRISPR/Cas9. The top panel shows 57 kb of DNA adjacent to the chromosome 12 telomere (at the right). The bottom panel is an enlargement of the *var2csa* gene (Pf3D7_1200600). DNA read enrichment is indicated on the *y* axis, and gene models are shown in gray at the bottom. A schematic of the positions of the telomere repeats (black “Tel”) and homology regions 1 and 2 (HR1 in green and HR2 in blue) within the telomere mutant is shown at the bottom. (C) Stranded sequencing of RNA harvested 12 hpi from WT 3D7 parasites (black), *var2csa* intron KO clone A (green), and a telomere mutant clone (red) showed that *var2csa* full-length transcripts were produced in 3D7 WT parasites and the intron KO clone. The telomere mutant clone produced *var2csa* transcript up to, but not past, the end of the second homology region used for CRISPR/Cas9 editing. Coverage plots are shown at the top of each panel, and read stacks are shown at the bottom, where red lines indicate sense transcription reads, and blue lines indicate antisense transcription reads. Numbers of reads are indicated on the *y* axis, and the gene model is shown in gray at the top.

All *var* genes have a similar structure consisting of a 5′ upstream promoter sequence followed by two exons that flank a conserved intron (Fig. 1A). Studies using reporter genes and drug-selectable markers have demonstrated that a “pairing” of the 5′ upstream promoter with the *var* intron is required for both silencing and maintenance of the counting mechanism involved in monoallelic expression (reviewed in reference 7). The 5′ upstream sequence constitutively drives expression of reporter genes; however, this promoter activity is silenced in an S-phase-dependent manner when a *var* intron is placed at the 3′ end of the reporter gene (8–10). In an episomal context, the ability of the *var* intron to silence transgene transcription depends on its own promoter activity, and pairing of a *var* 5′ upstream promoter sequence with any downstream active promoter is able to silence transgenes and maintain monoallelic expression (11, 12). However, it has been shown that transcriptional activation of an episomal or integrated *var* promoter is able to silence the members of the *var* gene family, suggesting that the *var* intron is not required for maintenance of monoallelic expression (13).

Interestingly, sense and antisense sterile transcripts have been shown to originate from the introns of *var* genes, suggesting that the *var* intron has bidirectional promoter activity that could be involved in transcriptional activation or silencing (Fig. 1A) (14–16). Recent studies provided evidence for a role of *var* intron-derived antisense long noncoding RNA (lncRNA) in *var* gene activation and monoallelic expression (3, 17, 18). However, microarray and RNA sequencing data from studies of endogenous *var* genes demonstrated that transcription originating from the *var* intron in either the sense or antisense direction showed no overall correlation with *var* gene activation or silencing (19, 20). Thus, it remains unclear if *var* intron-derived sterile transcripts are involved in *var* gene regulation (reviewed in reference 21).

An alternative, but perhaps related, mechanism of noncoding RNA-mediated *var* gene regulation is the specific binding of the intron to regulatory proteins. An 18-bp binding element present in the introns of a subset of *var* genes was shown to bind to the putative AP2 transcription factor and to actin (22). This intron element was able to localize episomes to the nuclear periphery, which is often associated with heterochromatin and transcriptional repression (5, 23, 24). Moreover, induction of actin polymerization led to the disruption of *var* gene localization and monoallelic expression (22). Thus, the *var* intron may mediate *var* gene transcription via binding to regulatory proteins and/or nuclear positioning.

The conflicting data supporting a role for the *var* intron in regulation of *var* gene transcription may be a consequence of the almost exclusive use of episomes, reporter genes, and selectable drug markers for the study of this genetic element. Because *var* gene transcription is regulated epigenetically, it is essential to study the underlying regulatory genetic elements in a natural, native chromatin context (25). To address the transcriptional role of the *var* intron, we used the recently developed clustered regularly interspaced short palindromic repeats (CRISPR)/Cas9 system (26) to directly delete the *var2csa* intron, producing an intronless *var* gene in its native locus without the introduction of a drug-selectable marker. We show that deletion of the *var2csa* intron leads to increased transcription of the *var2csa* gene (Pf3D7_1200600) in ring-stage parasites and can, in a minority of clones, lead to activation of transcription of other *var* genes. The intronless *var2csa* gene can be silenced in late-stage parasites and via long-term culture or panning with the CD36 receptor, although this silencing can be reversed via panning with chondroitin sulfate A (CSA). Our data suggest that, while the *var* intron is not required for silencing or activation of the *var2csa* gene, it may play a role in the transcriptional regulation and switching of the members of the *var* gene family.

## RESULTS

### Generation of the intronless *var* gene mutant.

To gain insight into the role of the *var* intron in *var* gene transcription regulation, we targeted the intron of the *upsE var2csa* gene in the *P. falciparum* 3D7 strain. Among the approximately 60 *var* genes found in the genomes of various *P. falciparum* isolates, *var2csa* is well conserved and is present even in the genome of the non-human primate parasite *P. reichenowi* (27). The expression of this particular *var* gene has been strongly implicated in the pathogenesis of placental malaria, as the resultant PfEMP1 protein binds strongly to CSA expressed on placental syncytiotrophoblasts (28–30). As the *var2csa* gene is unique and clinically relevant, it was ideal for our study because it was easily targeted by CRISPR/Cas9 and several useful reagents are available for studying its expression. However, the findings of our study might not necessarily apply to all subtypes of the *var* genes.

The *var2csa* gene is located in the subtelomeric region of chromosome 12 and consists of an 8,004-bp exon I, an 847-bp intron, and a 1,167-bp exon II (Fig. 1A, green, orange, and blue, respectively). We completely deleted the endogenous *var2csa* intron using the CRISPR/Cas9 genome-editing system recently described in reference 26. Cells were cotransfected with the pUF1_Cas9 and pL7_*var2csa*_IntKO plasmids (Fig. 1A). The Cas9 endonuclease was directed to the extreme 3′ end of exon I by a highly specific single guide RNA (sgRNA) designed with the newly developed Protospacer software (31). The resultant DNA double-strand break was repaired via homologous recombination with the intron knockout DNA construct provided on the pL7 plasmid. This construct consists of 500 bp of the 3′ end of exon I immediately upstream of the intron (Fig. 1A, homology region 1 in green) fused in frame to 500 bp of the 5′ end of exon II immediately downstream of the intron (Fig. 1A, homology region 2 in blue). The protospacer adjacent motif (PAM) sequence targeted by the sgRNA/Cas9 was mutated in the knockout construct to prevent further Cas9 endonuclease activity while conserving amino acid sequence (Fig. 1A, red star).

The result of the CRISPR/Cas9 process was an intronless *var2csa* gene: exon I fused in frame to exon II without the use of drug-selectable markers at the endogenous locus (Fig. 1A). After subcloning of the transfectants was performed, genomic DNA was prepared and the absence of the *var2csa* intron was confirmed with PCR and next-generation sequencing (Fig. 1A and B, respectively). Curiously, the vast majority of clones contained an unintended mutation in which endogenous DNA between the end of homology region 2 and the telomere was lost, leading to truncation of exon II (Fig. 1B, red). The sequencing data also revealed that in these telomere mutants, the Cas9-induced double-strand break resulted in chromosome breakage and repair with telomere repeats, a repair mechanism that has also been reported for spontaneous chromosome breakage in *in vitro* culture (32). While unexpected, the intronless *var2csa* telomere mutant clone served as a useful control for experiments discussed below. In the end, through genomic sequencing, we identified six clones from two independent transfections with correctly edited genomes. Representative genomic sequencing data are shown for clone A (Fig. 1B, green).

The *var* intron has been implicated in both silencing and activation of *var* gene transcription (reviewed in reference 7). To determine if the *var2csa* gene is transcribed in the absence of the intron, RNA was harvested from a highly synchronous 3D7 wild-type (WT) bulk culture, three *var2csa* intronless clones, and one *var2csa* intronless telomere mutant clone at 12 h post infection (hpi), a time when *var* genes are highly transcribed. Stranded RNA sequencing was performed using poly(A)-enriched RNA, confirming that mRNA containing exon I and exon II, but not the intron, is produced in WT and *var2csa* intronless mutant clones A, B, and C (Fig. 1C, WT in black and intron knockout [KO] mutant clone A in green). The intronless telomere mutant clone also transcribes *var2csa* but lacks all endogenous RNA sequence downstream of homology region II (Fig. 1C, in red).

### *var2csa* intron deletion results in upregulation of transcription in ring-stage parasites.

To determine if the *var* gene transcriptional profile is altered in the absence of the *var2csa* intron, we analyzed RNA levels of the ~60 *var* genes in the *var2csa* intronless mutants. A CSA-panned FCR3 culture, the 3D7 WT parent strain of the *var2csa* intronless mutants (bulk culture), one *var2csa* intronless telomere mutant clone, and six clones of the *var2csa* intron KO were tightly synchronized, and total RNA was harvested at 12 hpi (ring stage). Reverse transcription-quantitative PCR (RT-qPCR) was performed with primers specific to each of the ~60 *var* genes (from reference 28).

**FIG 2:**
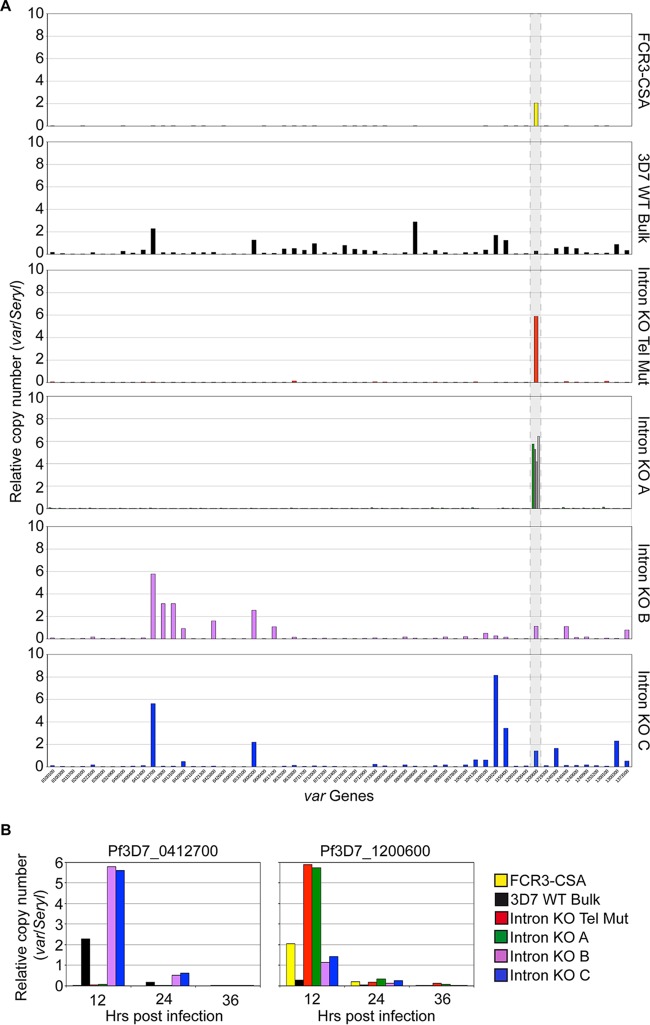
*var2csa* intron deletion results in upregulation of transcription in ring-stage parasites. (A) RNA was isolated from highly synchronized ring-stage parasites (12 hpi) from a CSA-panned FCR3 culture (top panel, yellow), a 3D7 WT bulk culture (second panel, black), an intron KO telomere mutant clone (third panel, red), and six *var2csa* intron KO mutant clones (fourth, fifth, and sixth panels). RT-qPCR analysis of all *var* genes was performed, and cDNA levels were normalized to those of serine-tRNA ligase. Intron KO clone A is shown in the fourth panel in green along with three other clones (in gray) showing similar monoallelic transcriptional *var* profiles. Intron KO clones B (fifth panel in purple) and C (sixth panel in blue) transcribed multiple *var* genes. A gray dashed box indicates the *var2csa* gene, Pf3D7_1200600. (B) RNA was isolated from highly synchronized parasites from a CSA-panned FCR3 culture (yellow); a 3D7 WT bulk culture (black); intron KO mutant clones A (green), B (purple), and C (blue); and an intron KO telomere mutant clone (red) at 12 (ring), 24 (trophozoite), and 36 (schizont) hpi. RT-qPCR analysis of Pf3D7_0412700 (*upsC var* gene) and Pf3D7_1200600 (*var2csa*) was performed, and *var* gene cDNA levels were normalized to those of serine-tRNA ligase.

As the FCR3 culture had been enriched for parasites expressing VAR2CSA, it predominantly transcribed the *var2csa* gene, or Pf3D7_1200600 (Fig. 2A, top panel in yellow). The 3D7 WT bulk parent culture was not clonal; thus, multiple different *var* genes, especially *upsC* types, were transcribed to various degrees in ring-stage parasites (Fig. 2A, second panel in black). The *var2csa* gene was not highly transcribed in the 3D7 culture; however, higher levels of *var2csa* transcription were seen in all intronless mutant clones (Fig. 2A, green/gray, purple, blue), including the telomere mutant (Fig. 2A, red).

In a clonal population of parasites, usually only a single *var* gene is transcribed (2). Indeed, transcription of a single *var* gene—the intronless *var2csa* gene—was observed in four different intron KO clones (Fig. 2A, fourth panel: intron KO clone A in green and three additional clones in gray) and the intron KO telomere mutant (Fig. 2A, third panel in red). However, intron KO clones B (purple) and C (blue) transcribed distinct subsets of *var* genes, both subtelomeric and central, in addition to *var2csa* (Fig. 2A, fifth and sixth panels). The latter pattern of transcription suggests loss of monoallelic expression or an increase in *var* gene switching within the clonal population. These data were supported by the stranded RNA sequencing analysis performed for intron KO clones A, B, and C and the telomere mutant clone.

RT-qPCR analysis was repeated with RNA harvested at 24 and 36 hpi, when *var* gene transcription is normally repressed, using primers against the Pf3D7_0412700 central *var* gene and the Pf3D7_1200600 *var* gene. In 3D7 WT bulk culture parasites and *var2csa* intron KO clones B and C, both the Pf3D7_0412700 and Pf3D7_1200600 *var* genes were transcribed at 12 hpi but at background levels at 24 and 36 hpi (Fig. 2B, left and right panels, respectively). Similarly, the Pf3D7_1200600 *var* gene was transcribed in FCR3-CSA; *var2csa* intron KO clones A, B, and C; and the *var2csa* intronless telomere mutant clone at 12 hpi but was transcriptionally silenced in later-stage parasites (Fig. 2B, right panel). Together, these data suggest that the intron is not required for *var2csa* gene activation in rings or silencing in trophozoites and schizonts but may be involved in switching of the *var* gene family.

### A *var2csa* intronless mutant produces VAR2CSA PfEMP1 protein.

The VAR2CSA PfEMP1 binds to the proteoglycan CSA, which is highly expressed on the surface of placental syncytiotrophoblasts (29, 30). It has been previously reported that VAR2CSA expression is regulated at the translational level, as *var2csa* mRNA is sometimes expressed in infected individuals and cultured parasites without detectable VAR2CSA PfEMP1 expression (33–35). This translational regulation is believed to be controlled by an open reading frame upstream of the actual *var2csa* open reading frame (36, 37). To determine if the *var2csa* intron knockout strains produce PfEMP1, we performed Western blot analysis and immunofluorescence assay (IFA).

**FIG 3:**
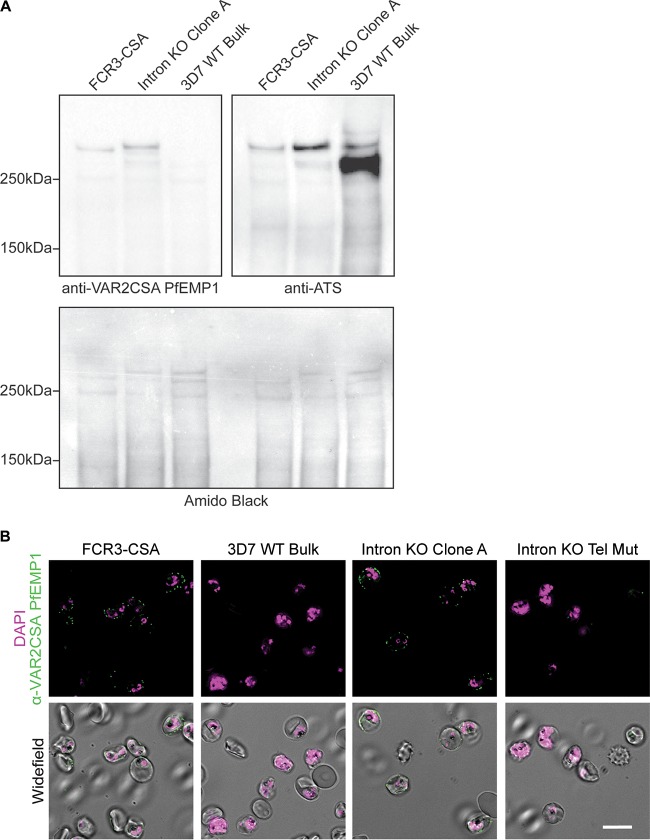
*var2csa* intronless mutant produces VAR2CSA PfEMP1 protein. (A) Western blot analysis of 30-hpi iRBC membrane extracts performed using antibodies against VAR2CSA PfEMP1 (top left panel) or the PfEMP1 ATS region (top right panel). Extracts were prepared from a CSA-panned FCR3 culture, intron KO clone A, and a 3D7 WT bulk culture. An amido black staining of the Western blot is shown as a loading control. Molecular masses are indicated at the left. (B) Immunofluorescence analysis of live 30-hpi iRBCs containing CSA-panned FCR3, 3D7 WT bulk, intron KO clone A, or intron KO telomere mutant clone parasites. DNA was stained with DAPI (magenta), and iRBCs were surface labeled with specific antibodies against VAR2CSA PfEMP1 (green). Fluorescent images are shown in the top panel, and wide-field merged images are shown in the bottom panel. Scale bar, 10 μm.

Western blot analysis of 30-hpi iRBC membrane fractions with an antibody against the VAR2CSA PfEMP1 revealed that *var2csa* intron knockout clone A produces VAR2CSA at levels that are similar to, if not higher than, those of VAR2CSA produced by a CSA-panned FCR3 bulk culture (Fig. 3A). VAR2CSA expression is undetectable in the bulk 3D7 parent strain of the *var2csa* intron knockout, although expression of other PfEMP1 proteins can be detected with an antibody against the acidic transmembrane segment (ATS), which is highly similar among all PfEMP1 proteins (Fig. 3A).

A similar pattern of VAR2CSA expression was seen with surface IFA of live 30-hpi iRBCs using DAPI (4′,6-diamidino-2-phenylindole) and the antibody against VAR2CSA PfEMP1. The CSA-panned FCR3 culture showed expression of VAR2CSA at the membrane of approximately 90% iRBCs, while *var2csa* intron KO clone A showed expression of VAR2CSA at the membrane of approximately 80% iRBCs (Fig. 2B). VAR2CSA was present in a small percentage of 3D7 WT iRBCs but was undetectable by IFA in the *var2csa* intron KO telomere mutant clone (Fig. 2B) and also in *var2csa* intron KO clone C. These data suggest that the intron is not needed for proper mRNA processing or translation but support previous studies demonstrating VAR2CSA PfEMP1 expression control at the translational level (36, 37).

### An intronless *var2csa* gene can be silenced via long-term culture or CD36 panning, then reactivated with CSA panning.

To determine if the intron is required for *var* gene silencing, we attempted to induce the *var2csa* intronless mutant parasites to switch *var* transcription in a natural manner that did not involve the use of episomes. It has been shown that *var* gene switching occurs spontaneously when a parasite population is kept in culture for long periods of time, especially in the case of subtelomeric *var* genes (38). Switching can also be induced by selective binding, or panning, of iRBCs to various host receptors. As many as 84% of PfEMP1 proteins—excluding VAR2CSA—are believed to bind to human endothelial receptor CD36 (1). Thus, we attempted to induce *var* gene switching via long-term culture and panning with CD36. A 3D7 bulk culture and *var2csa* intron KO clone A were tightly synchronized, and RT-qPCR was performed on RNA harvested at 12 hpi using primers against all ~60 *var* genes. These cultures were then split and kept in continuous culture for 2 months or were used to perform three rounds of CD36 panning, after which we confirmed maintenance of the intron knockout mutation with PCR and sequencing. RNA analysis was then repeated with synchronized parasites.

**FIG 4:**
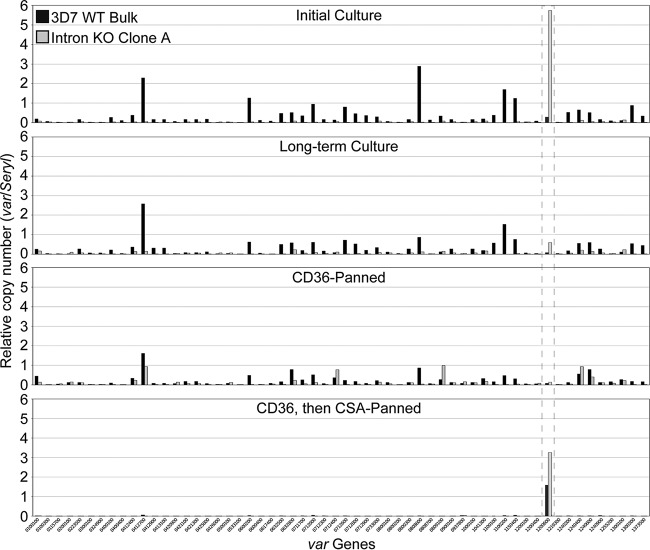
An intronless *var2csa* gene can be silenced via long-term culture or CD36 panning, then reactivated with CSA panning. RNA was isolated from highly synchronized ring-stage parasites (12 hpi) from a 3D7 WT bulk culture (black) and *var2csa* intron KO mutant clone A (gray). RT-qPCR analysis of all *var* genes was performed before (“Initial Culture,” top panel) and after (“Long-term Culture,” second panel) 2 months of continuous culture. The initial cultures were also subjected to three rounds of CD36 panning (third panel), followed by three rounds of CSA panning (bottom panel). *var* gene cDNA levels are normalized to those of serine-tRNA ligase. A gray dotted box indicates the *var2csa* gene, Pf3D7_1200600.

The transcript levels of several different *var* genes changed noticeably between the initial time point (Fig. 4, top panel) and 2 months in culture (Fig. 4, second panel) or CD36 panning (Fig. 4, third panel) in the 3D7 bulk culture and the *var2csa* intron KO clone (Fig. 4, black and gray bars, respectively). Notably, *var2csa* transcription was reduced substantially in the *var2csa* intron KO clone after 2 months of continuous culture and was barely detectable after CD36 panning.

To determine if the intronless *var2csa* gene could be transcriptionally reactivated once silenced, we performed three rounds of CSA panning with the CD36-panned cultures. While three rounds of CSA panning were required to observe adherence of the 3D7 bulk culture to CSA, the *var2csa* intron KO culture adhered well after just one round of CSA panning. These panned cultures were synchronized, and *var* transcriptional analysis was carried out at 12 hpi. Indeed, the transcriptional profile demonstrates almost exclusive transcription of the *var2csa* gene in both strains, although it was transcribed to a higher degree by the *var2csa* intron KO culture (Fig. 4, bottom panel). These data further demonstrate that the intron is not required for *var2csa* transcriptional silencing, activation, or switching and that the lack of the intron perhaps allows easier transcriptional activation and switching of the *var2csa* gene.

## DISCUSSION

In *P. falciparum*, *var* gene transcription is under tight control. Although the 5′ upstream promoter and intron have been implicated in this process, the true role of these genetic elements has been difficult to elucidate, as most previous studies have relied on episomes and/or transgenes. Using the CRISPR/Cas9 system, we directly deleted a *var* intron without insertion of a drug-selectable marker at the endogenous locus. Although previous studies of *var* gene transcription have primarily used genetic elements of central *var* genes, we focused our study on the subtelomeric Pf3D7_1200600 (*var2csa*) gene, as this *var* gene is well conserved across many *P. falciparum* strains and has been strongly implicated in the pathogenesis of pregnancy-associated malaria (reviewed in reference 39).

Our data show that deletion of the *var2csa* intron leads to transcriptional upregulation of the targeted *var2csa* gene in ring-stage parasites (Fig. 1C and 2A). These data might suggest that the intron is required for *var2csa* transcriptional silencing, but we show that transcription of the intronless *var2csa* gene could be downregulated in late-stage parasites and in response to long-term culture or CD36 panning (Fig. 2B and 4). It is possible that the intron is needed for *var2csa* gene activation and that the initial increase in transcription of the intronless *var2csa* gene was the result of transcriptionally activating epigenetic changes taking place in response to the Cas9-induced DNA damage and repair rather than to the loss of an intron-binding silencing factor. However, we show that transcription of the intronless *var2csa* gene can be easily reactivated by CSA panning after being silenced by CD36 panning (Fig. 4).

Thus, in the case of *var2csa*, the intron is not required for transcriptional silencing or activation. Our data do not support earlier studies that used episomes and transgenes to demonstrate the requirement of the *var* intron in silencing the *var* 5′ UTR promoter (8–10). Conflicting reports have provided evidence that antisense and/or sense sterile transcripts originating from the intron play a role in both *var* gene silencing and activation (3, 14, 17–19). However, our RT-qPCR and RNA sequencing data demonstrate that the *var* intron and any sense or antisense sterile transcripts that originate from the intron are not strictly required for *var2csa* gene activation or silencing (Fig. 1C, 2A, and 4). It is possible, however, that these intron-based mechanisms may modulate *var* gene activation or silencing for other *var* subtypes and maybe even switching or counting for *var2csa*. Given that functional differences between different *var* introns were previously observed (22), it is possible that some introns have distinct roles in the control of mutually exclusive *var* gene transcription.

Our data do support previous studies showing that PfEMP1 protein expression is not required for monoallelic expression, as multiple clones of the intron KO telomere mutant transcribed only the targeted *var2csa* gene in the absence of functional VAR2CSA PfEMP1 (Fig. 2A, red) (40). Previous studies have shown that VAR2CSA PfEMP1 expression is subject to posttranscriptional regulation (35–37). Our data support these studies, as we saw a lack of VAR2CSA PfEMP1 expression via IFA in intron KO clone C, which clearly transcribed *var2csa* (Fig. 2A [blue] and data not shown). It is unclear, however, which of the many *var* genes transcribed in these intron KO clones are expressed at the protein level, as specific antibodies are currently unavailable.

This report provides new insight into the plastic nature of intron-mediated *var* gene switching and substantiates the complexity of *var* gene transcriptional regulation in the chromosomal context. In our study, different transcriptional phenotypes were seen in different genetically identical mutant clones, as two of six intron KO clones transcribed distinct sets of multiple *var* genes (Fig. 2A, purple and blue). Considering that transcription of a single *var* gene is usually stable over the course of cloning and synchronization in a WT clonal population of parasites, we postulate that our mutant clones have an increased rate of *var* gene switching. It has been shown that *P. falciparum* cultures tend to switch preferentially from transcription of subtelomeric *var* genes to transcription of central *var* genes and that *var2csa* may play a unique coordinating role in *var* gene switching, serving as a default switching node (38, 41). Interestingly, half of the *var* genes that were transcribed in addition to the intronless *var2csa* in our two mutant clones were subtelomeric *var* genes, and none were adjacent to the *var2csa* gene (Fig. 2A, purple and blue). This observation might hint at an intron-based telomeric clustering mechanism used to silence *var* genes and/or to coordinate *var* gene switching in *trans*, as has been suggested previously (5). It is possible that putative repressive *var* gene clusters are controlled by a delicate balance of intron-binding proteins or associated ncRNAs, and it will be critical to identify these potential regulators in the future.

The *var2csa* gene has been shown to be a unique case among *var* genes, being subject to transcriptional and posttranscriptional control. Thus, it remains to be seen if intron deletion in other *var* subtypes would result in a phenotype similar to that of *var2csa*. We attempted to create several different intron knockouts in *upsA*, *upsB*, and *upsC var* genes but were unsuccessful due to chromosomal aberrations resulting from Cas9-induced DNA damage. Each time we attempted to delete the intron from a different subtelomeric *var* gene, close to 100% of the transfected parasite population acquired the same telomeric mutation that we saw to a lesser extent in the *var2csa* intron KO mutants. Moreover, induced DNA damage occurring with any type of *var* gene may lead to interchromosomal recombination events, a mechanism that *P. falciparum* is known to use to survive *in vivo*. Thus, the CRISPR/Cas9 system may be challenging to use in the context of the subtelomeric or even central *var* genes, and we recommend caution and genomic sequencing for any mutant created with CRISPR/Cas9.

## MATERIALS AND METHODS

### Parasite culture.

Asexual blood-stage *P. falciparum* parasites were cultured as previously described in reference 5. Parasites were synchronized by sorbitol lysis at the ring stage, by Plasmagel enrichment 24 h later, and by an additional sorbitol lysis 6 h after Plasmagel enrichment. Parasite development was monitored by Giemsa staining. Parasites were harvested at 4% hematocrit and approximately 5% parasitemia.

### Strain creation.

*var* intron knockout parasites were created from the 3D7 wild-type strain using the CRISPR/Cas9 system as previously described in reference 26. For *var2csa* (Pf3D7_1200600), a 20-nucleotide sgRNA (GGTTTTTTGCAGAATGTCAC) was designed using Protospacer software (31) and inserted into the pL6 sgRNA expression plasmid. To facilitate knockout via homologous recombination, homology regions were ordered from GenScript, PCR amplified, and inserted into the same pL6 plasmid. Homology regions were designed by fusion of a 500-bp sequence of exon I directly upstream of the intron to a 500-bp sequence of exon II directly downstream of the intron. Silent shield mutations were introduced into and near the protospacer adjacent motif sequence of the homology region (GGTTTTTTGCAGAATGTCGCTCG [the mutations are underlined]). All cloning was performed using HiFi DNA polymerase (Kapa Biosystems), an In-Fusion HD cloning kit (Clontech), and XL10-Gold ultracompetent *Escherichia coli* (Agilent Technologies). Ring-stage parasites were transfected with pUF1-Cas9 and pL7 plasmids as previously described in reference 26. Cloning of parasites was carried out by limiting dilution.

### Genomic DNA preparation, PCR, and sequencing.

Genomic DNA was isolated with a DNeasy Blood and Tissue kit (Qiagen) from late-stage parasites isolated with manual cell separation (MACS) columns (Miltenyi Biotec) or Plasmagel. For sequencing, purified genomic DNA was sheared with a Bioruptor standard ultrasonicator (Diagenode). Libraries were prepared for sequencing using MicroPlex Library Preparation kit v2 (Diagenode). Libraries were sequenced on a NextSeq 500 or HiSeq platform (Illumina). Sequenced reads were mapped to the *P. falciparum* genome (42) (plasmoDB.org, version 3, release 29) using “bwa mem” (43) and allowing a read to align only once to the reference genome (option “-c 1”). Alignments were subsequently filtered for duplicates and a mapping quality value of ≥30 using samtools (44) and visualized in the Integrative Genomics Viewer (45).

### RNA extraction and qPCR analysis.

Total RNA was isolated from synchronized parasite cultures by saponin lysis followed by purification performed with an miRNeasy kit (Qiagen). RNA was subjected to DNase (Qiagen) treatment, and reverse transcription was performed using random hexamer primers and SuperScript VILO reverse transcriptase (Thermo Fisher Scientific). Quantitative PCR was performed with the resultant cDNA in triplicate on a CFX real-time PCR system (BioRad) using Power SYBR green (Life Technologies, Inc.) and primers from a previous study (28). Transcript levels were determined using the quantity mean determined for each triplicate as calculated from a standard curve based on a serial dilution of genomic DNA. *var* gene transcription was normalized to that of a housekeeping gene, the serine-tRNA ligase gene (PF3D7_0717700).

### Stranded RNA sequencing and analysis.

Total RNA was extracted as described above. RNA samples were depleted of rRNA by the use of a Dynabeads mRNA Direct kit (Ambion 61012), and libraries were prepared with a TruSeq Stranded mRNA LT Sample Prep kit (Illumina). Libraries were sequenced on a NextSeq 500 platform (Illumina). Sequenced RNA reads were mapped, aligned, and visualized as described above for the genomic DNA.

### Western blot analysis.

iRBCs containing synchronous parasites were isolated at 30 hpi by Plasmagel enrichment. iRBCs were washed once with phosphate-buffered saline (PBS), and soluble proteins were extracted with NETT buffer (50 mM Tris [pH 8], 150 mM NaCl, 5 mM EDTA, 1% Triton X-100) containing protease inhibitors (Roche 11836170001). This extract was centrifuged at 16,000 relative centrifugal force (rcf), and the membrane fraction was extracted with Tris-saline buffer (50 mM Tris [pH 8], 150 mM NaCl, 2% SDS) containing protease inhibitors (Roche 11836170001). The extract was sonicated for 5 min (30 s on, 30 s off) with a Diagenode Bioruptor and then centrifuged at 16,000 rcf. Proteins in the supernatant were separated on a 3% to 8% Tris-acetate NuPage gel and transferred to a polyvinylidene difluoride (PVDF) membrane. The membrane was blocked for an hour with 5% milk–TBST (50 mM Tris, 150 mM NaCl, 0.1% Tween 20). PfEMP1 proteins were detected with guinea pig anti-PfEMP1 acidic transmembrane segment (ATS) (46) and rabbit anti-VAR2CSA PfEMP1 (47) primary antibodies, followed by goat anti-guinea pig horseradish peroxidase (HRP) (Abcam, Inc.; ab6908) and donkey anti-rabbit HRP (GE Healthcare Life Sciences; NA934V) secondary antibodies. HRP signal was developed with SuperSignal West Pico chemiluminescent substrate (Thermo Fisher Scientific; 34080) and imaged with a ChemiDoc XRS+ system (Bio-Rad).

### Immunofluorescence assay.

Prior to labeling, polyclonal rabbit anti-VAR2CSA antibody was diluted 1:500 and subjected to preadsorption with 10 µl uninfected red blood cells for 1 h in 4% bovine serum albumin (BSA)–PBS. A Plasmagel flotation assay was performed on parasite culture to enrich for late-stage iRBCs. After washing 10 µl of iRBCs with PBS, cells were blocked with 4% BSA–PBS for 30 min, followed by incubation with 50 µl preadsorped primary antibody dilution for 1 h, three washes with PBS, and detection with Alexa Fluor 488-conjugated anti-rabbit IgG (Invitrogen) diluted 1:1,000 in 4% BSA–PBS. After three final washes in PBS, cells were mounted in Vectashield containing DAPI for nuclear staining. Images were captured using a Nikon Eclipse 80i microscope with a CoolSnap HQ2 camera (Photometrics). NIS elements 3.0 software (Nikon) was used for acquisition and Fiji software (http://fiji.sc/) for analysis.

### Receptor panning.

Plastic cell culture dishes were coated with CD36 receptor (Life Technologies, Inc.; 10752-H08H) (4 µg/ml PBS) or CSA (Sigma; C9819) (1 mg/ml PBS) overnight at 4°C. The dishes were blocked with 1% BSA (Sigma, A4503)–PBS for 1 h at 37°C. iRBCs containing trophozoites and schizonts were isolated by Plasmagel enrichment and resuspended in 8 ml binding medium (RPMI 1640 with 25 mM HEPES [pH 6.8 for CD36 and pH 7.2 for CSA]) at a concentration of 5 × 10^7^ iRBCs/ml. After the dish was washed once with binding medium, iRBCs were added and allowed to adhere for 1 h with gentle agitation at 37°C. The dish was washed with binding medium to eliminate all unbound cells, and the bound cells were recovered with normal medium. The culture was allowed to recover, and panning was repeated twice more. Binding was assessed at each panning by microscope.

### Data availability.

Genomic DNA (3D7 WT clone, intron KO clone A, and intron KO telomere mutant clone) and RNA (3D7 WT bulk, intron KO clone A, and intron KO telomere mutant clone) sequencing fastq files are available from the National Centre for Biotechnology Information’s Sequence Read Archive (SRA) under project accession number PRJNA385099.
